# Rational Search for Betaine/GABA Transporter 1 Inhibitors—*In Vitro* Evaluation of Selected Hit Compound

**DOI:** 10.1021/acschemneuro.4c00425

**Published:** 2024-10-19

**Authors:** Kamil Łątka, Stefanie Kickinger, Zuzanna Rzepka, Paula Zaręba, Gniewomir Latacz, Agata Siwek, Małgorzata Wolak, Dorota Stary, Monika Marcinkowska, Petrine Wellendorph, Dorota Wrześniok, Marek Bajda

**Affiliations:** † Department of Physicochemical Drug Analysis, Jagiellonian University Medical College, Medyczna 9, 30-688 Kraków, Poland; ‡ Department of Drug Design and Pharmacology, Faculty of Health and Medical Sciences, University of Copenhagen, 2100 Copenhagen, Denmark; § Department of Pharmaceutical Chemistry, Faculty of Pharmaceutical Sciences in Sosnowiec, Medical University of Silesia, Jagiellońska 4, 41-200 Sosnowiec, Poland; ∥ Department of Technology and Biotechnology of Drugs, Jagiellonian University Medical College, Medyczna 9, 30-688 Kraków, Poland; ⊥ Department of Pharmacobiology, Faculty of Pharmacy, Jagiellonian University Medical College, Medyczna 9, 30-688 Kraków, Poland; # Department of Medicinal Chemistry, Jagiellonian University Medical College, Medyczna 9, 30-688 Kraków, Poland

**Keywords:** betaine/GABA transporter 1, inhibitor, virtual screening, biological evaluation, rigid GABA analogue

## Abstract

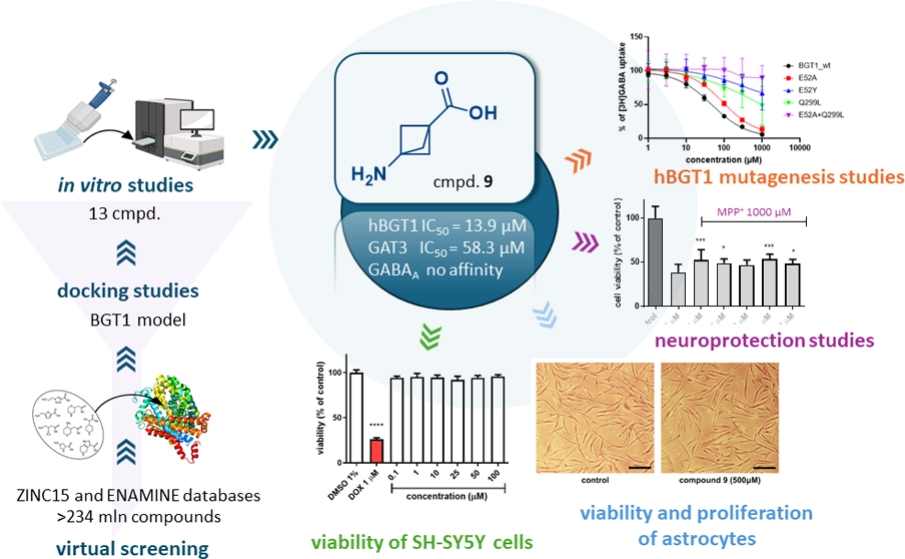

Inhibitory neurotransmission mediated by γ-aminobutyric acid (GABA) plays an important role in maintaining body homeostasis. Disturbances in GABA signaling are implicated in a multitude of neurologic and psychiatric conditions, including epilepsy, ischemia, anxiety, depression, insomnia, and mood disorders. Clinically relevant increases in GABA neurotransmitter level can be achieved by inhibition of its uptake into presynaptic neurons and surrounding glial cells, driven by GABA transporters (GAT1, BGT1, GAT2, and GAT3). Herein, we focused on the search for inhibitors of the BGT1 transporter which is understudied and for which the therapeutic potential of its inhibition is partly unknown. We applied multilevel virtual screening to identify compounds with inhibitory properties. Among selected hits, compound **9** was shown to be a preferential inhibitor of BGT1 (IC_50_ 13.9 μM). The compound also revealed some inhibitory activity against GAT3 (4x lower) while showing no or low activity (IC_50_ > 100 μM) toward GAT1 and GAT2, respectively. The predicted binding mode of compound **9** was confirmed by mutagenesis studies on E52A, E52Y, Q299L, and E52A+Q299L human BGT1 mutants. Subsequent evaluation showed that the selected hit displayed no affinity toward major GABA_A_ receptor subtypes. Moreover, it was nontoxic when tested on normal human astrocytes and even showed some neuroprotective activity in SH-SY5Y cells. Compound **9** is considered a promising candidate for further evaluation of the therapeutic potential of BGT1 transporter inhibition and the development of novel inhibitors.

## 1 Introduction

γ-Aminobutyric acid (GABA) is the main inhibitory neurotransmitter in the central nervous system (CNS).^1^ It plays a significant role in maintaining the balance between excitatory and inhibitory neurotransmission which is extremely important for proper functioning of the CNS.^2^ Disturbances in this balance are reported in several neurological disorders, such as epilepsy, insomnia, Alzheimer’s disease, and ischemia.^3^ The inhibitory action of GABA is achieved by GABA receptors that are categorized into the GABA_A_ and GABA_B_ receptor types.^2^ The main pharmacological effect of GABA signaling is predominantly connected to GABA_A_ receptors. Those located synaptically are responsible for phasic inhibition, while those located extrasynaptically cause persistent tonic inhibition.^4^ The extracellular level of GABA is regulated by GABA transporters (GATs) that facilitate the uptake of neurotransmitters into presynaptic neurons and surrounding glial cells.^4^ Synaptic spillover may then lead to relevant ambient GABA levels at extrasynaptic sites too.^5^ GATs belong to the solute carrier 6 (SLC6) family. According to the International Union of Basic and Clinical Pharmacology, in humans, these sodium-dependent transporters can be divided into four types: GAT1, BGT1 (betaine/GABA transporter 1), GAT2, and GAT3.^6^ GAT1 is primarily located in neurons, while GAT3 is mostly expressed in astrocytes. Even though BGT1 and GAT2 are abundantly expressed in the liver and the kidneys, they are also present in the brain, however at a low level.^3,7^ For BGT1, the subcellular distribution is predominantly limited to glial cells, suggesting a role in the regulation of extrasynaptic GABA levels. Broad studies on inhibition of the transporters, especially GAT1, resulted in the development of tiagabine (GAT1 inhibitor) which has been the only registered drug targeting GABA transport so far.^8−10^ This anticonvulsant drug is not devoid of substantial adverse reactions, such as asthenia, dizziness, nervousness, and depression.^8^ Therefore, the search for antiepileptic agents also includes other GABA transporters as therapeutic targets. Although the BGT1 transporter was found to be expressed at low levels under normal conditions, its inhibitors were proposed to have some anticonvulsant potential in certain forms of epilepsy.^3,7^ The most potent and selective BGT1 inhibitor is the rigid GABA analog—bicyclo-GABA ((1S,2S,5R)-5-aminobicyclo[3.1.0]hexane-2-carboxylic acid) with submicromolar/micromolar activity (IC_50_ = 0.59 μM/1.5 μM).^11,12^ It has not been tested *in vivo* so far, and thus, its antiepileptic effect remains unknown. On the other hand, the BGT1/GAT1 mixed inhibitor EF1502 and the BGT1 selective inhibitor RPC-425 were found to display anticonvulsant properties in mouse models.^13^ RPC-425 coadministered with tiagabine revealed an additive anticonvulsant effect, while EF1502 showed a synergistic effect.^14^ Thus, extrasynaptic BGT1 is suggested to play some role in epilepsy, however, still somewhat elusive as BGT1 knockout mice presented unchanged seizure threshold.^13^ Besides regulating GABA levels, BGT1 is also responsible for osmoregulation in the kidneys and delivery of betaine as a methyl donor for metabolic processes in liver.^7^ To determine the role of BGT1, especially in the CNS, better tool compounds are needed. These compounds should be characterized by high activity and selectivity. Moreover, they should penetrate the blood–brain barrier to make studies on CNS effects feasible.^14^

For the purpose of this study, we herein applied the virtual screening of compound databases to find new BGT1 inhibitors. After initial screening, the most promising compound (**9**) was subjected to extended pharmacological evaluation where activities against all subtypes of GABA transporters were determined, and the predicted binding mode to BGT1 was confirmed by site-directed mutagenesis studies. As GABA_A_ receptors are potential targets of GABA-like GAT inhibitors, compound **9** was also assessed for affinity to these receptors to most precisely describe the pharmacological profile. Finally, to test under native conditions, the impact of inhibitor **9** on the viability and proliferation of normal human astrocytes was evaluated, and the neuroprotective activity in the SH-SY5Y neuroblastoma cell line was determined.

## 2 Results and Discussion

### 2.1 Virtual Screening

**Figure 1:**
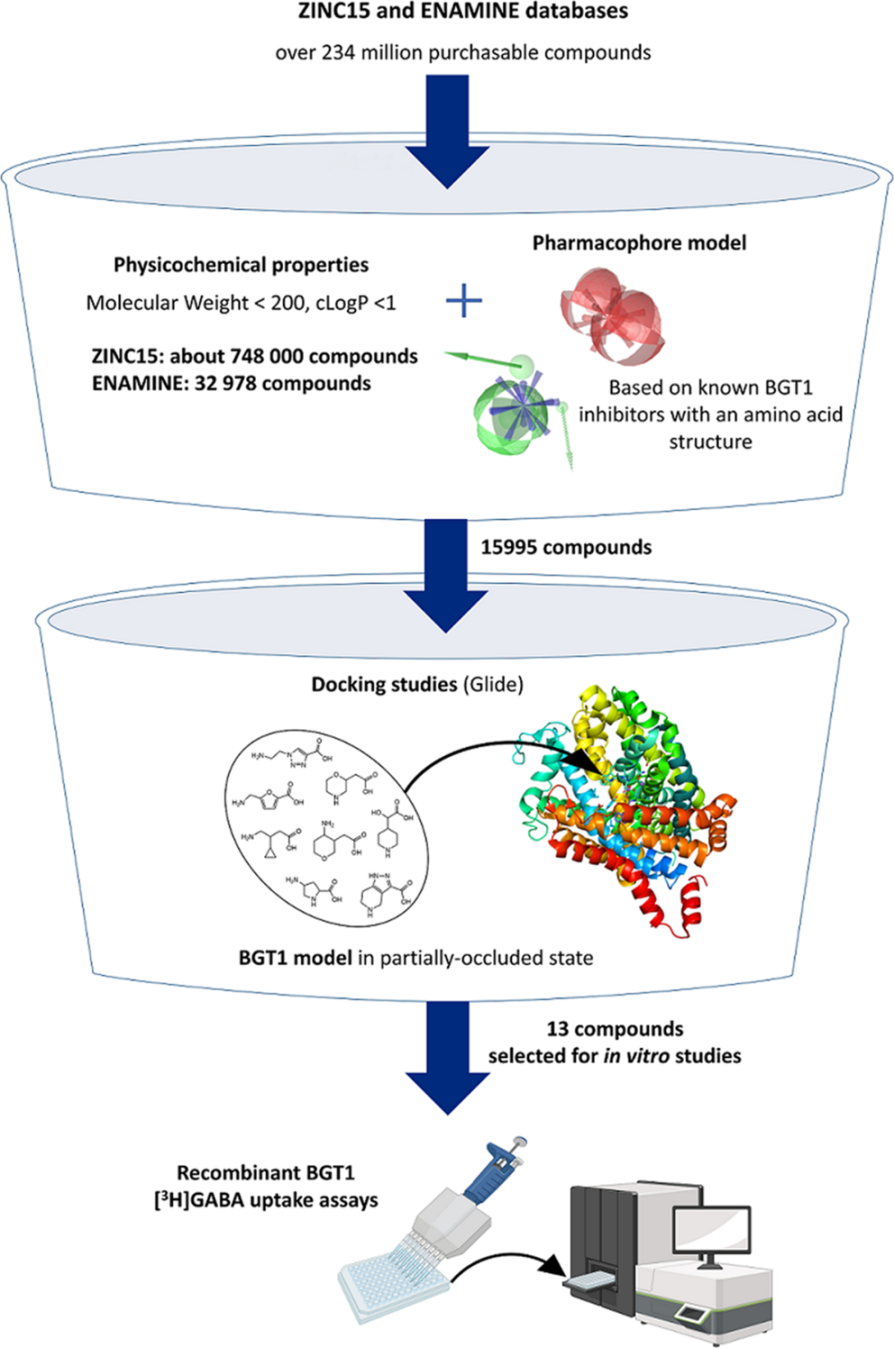
Virtual screening of ZINC15 and ENAMINE databases in the search of novel BGT1 inhibitors.

**Figure 2:**
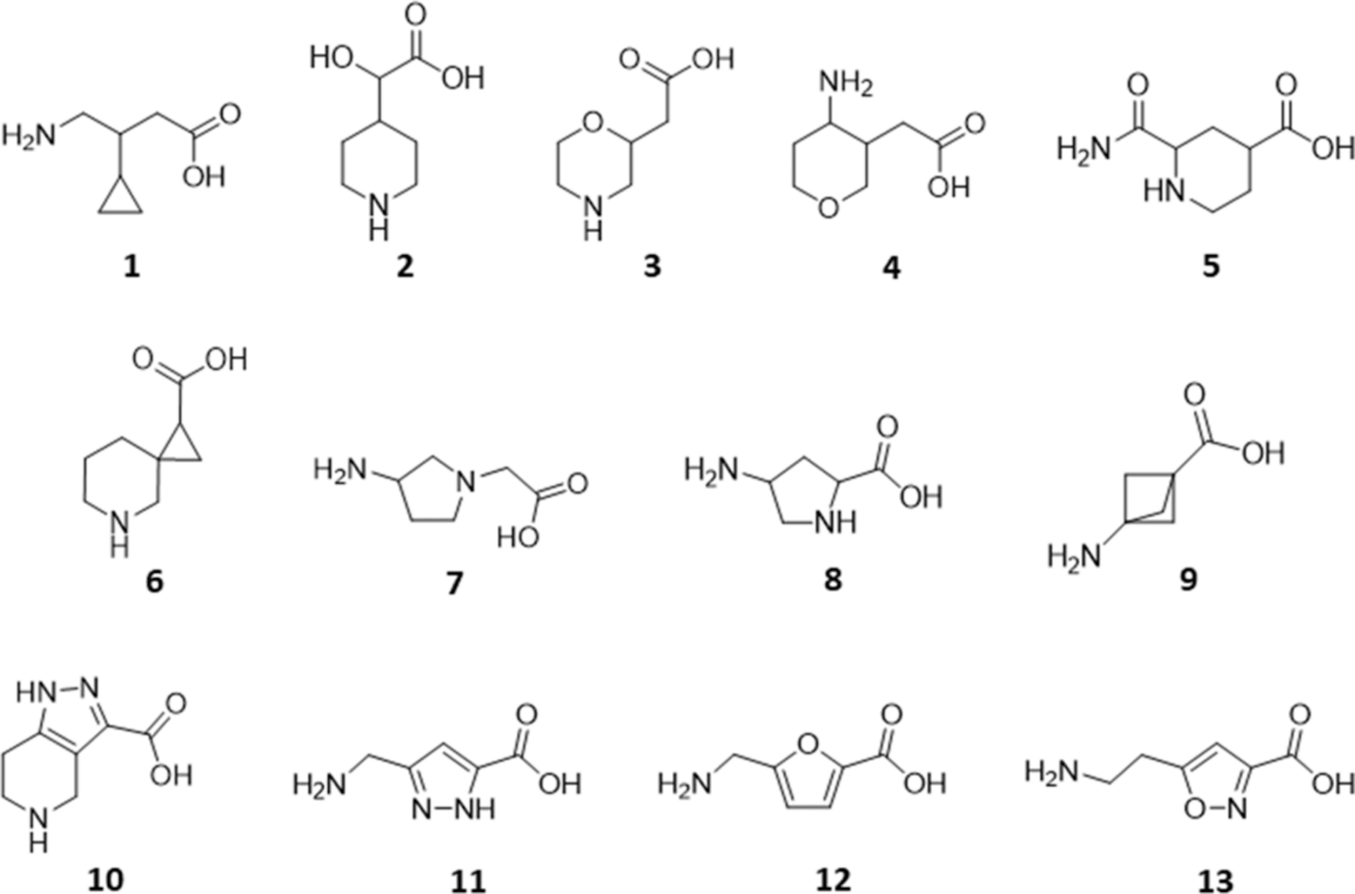
Compounds were selected from virtual screening.

The search for new BGT1 inhibitors involved screening of the ZINC15 and Enamine Screening Collection databases, which contain more than 230 million and 4 million commercially available compounds, respectively.^15^ In the first step, a physicochemical filter was used to select compounds with a molecular weight below 200 and cLogP below 1. The cutoff values were determined based on the properties of the most active small-molecule amino acid inhibitors. Such properties increase the likelihood of finding active compounds and provide broader opportunities for further optimization to obtain orally available CNS-penetrating inhibitors. This yielded 748,000 structures from ZINC15 and 32,978 from the Enamine databases (Figure 1). A pharmacophore model based on known amino acid BGT1 inhibitors (Supporting Information) was then applied. It consisted of seven molecular features: positive ionizable area; negative ionizable area; three hydrogen bond donors; and two hydrogen bond acceptors. Those compounds were selected that fulfilled at least five features, including negative and positive ionizable areas, and demonstrated an acceptable fit to the pharmacophore, expressed by the score value greater than 57.58, corresponding to the least potent of the active compounds. Using this approach, 13,861 structures from ZINC15 and 2134 from ENAMINE were chosen and then docked into a prebuilt human BGT1 (hBGT1) homology model.^16^ The model was based on the crystal structure of the dopamine transporter (PDB code: 4XPA) in the partially outward-occluded state. It was assumed that compounds similar in size and structure to the endogenous substrate should fit into this transporter conformation. Considering the recent GAT1 structures as alternative templates,^17−19^ the fact that they describe GAT1 in mainly the inward-open state made them less suitable to build the models for our virtual screening as only larger compounds were shown to bind. Furthermore, the deposited AlphaFold model of BGT1 revealed several less reliable structural features (position of Trp60 side chain or folding of Ser456-Ser457-Gly458 fragment) and was therefore omitted. The docked compounds were ranked according to their docking score and from the top 10%, the final 13 compounds were selected (10 compounds from ZINC15, 3 compounds from ENAMINE), taking into account binding mode, especially interactions with Gly57, Glu52, and Gln299, as well as availability and cost of the compounds (Figure 2).

### 2.2 *In Vitro* Assays for GABA Transport Inhibition

**Figure 3:**
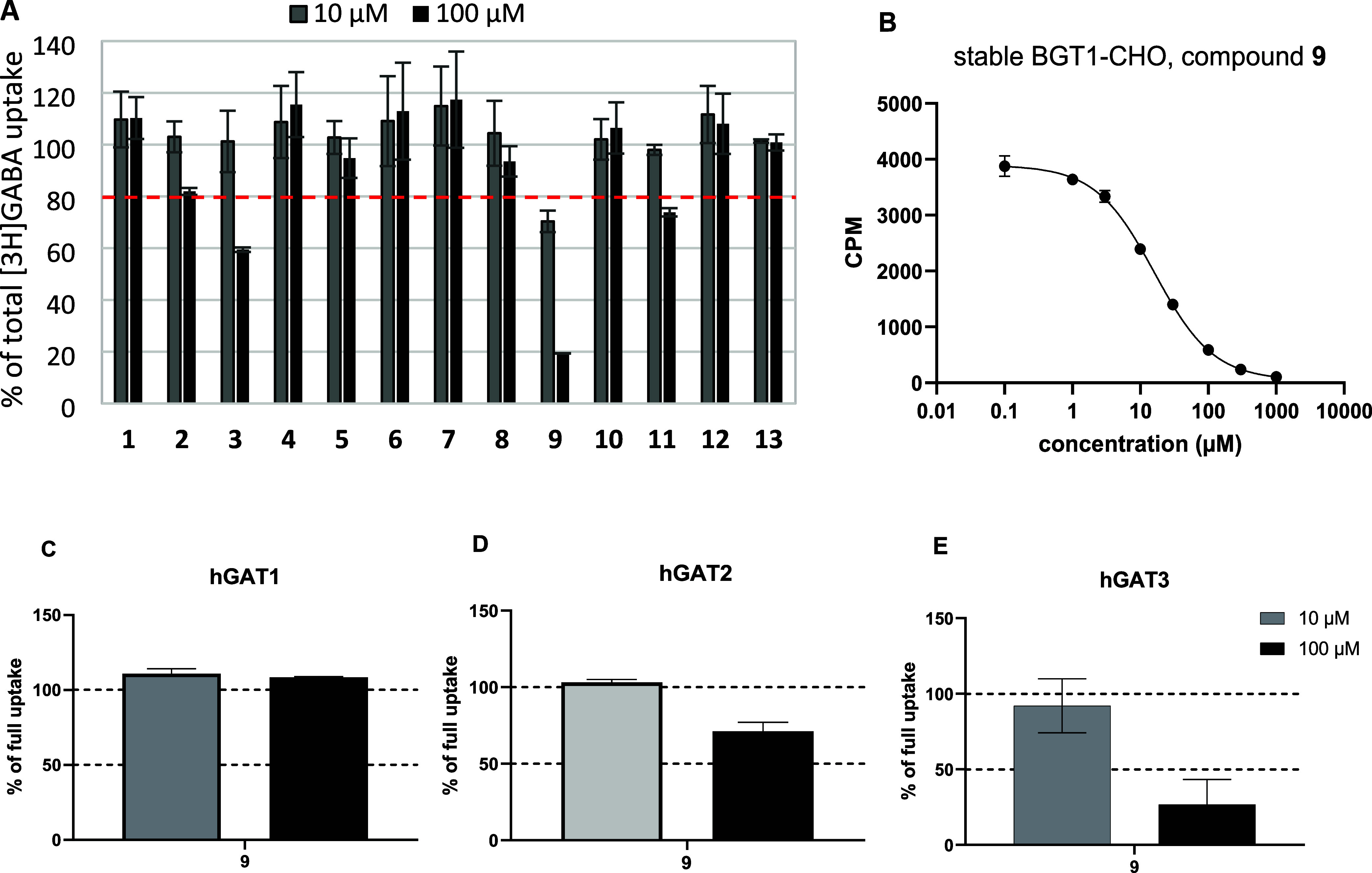
*In vitro* screening results for selected compounds are presented as a percentage of total [^3^H]GABA uptake by hBGT1 stably expressed in CHO cells (A). Compounds were tested at 10 and 100 μM concentrations. Data shown are pooled experiments (*n* = 2) with three technical replicates normalized to 100% [^3^H]GABA uptake (mean ± SEM). IC_50_ determination for the most active compound **9** (B). Data represent one of three independent experiments, each performed in three technical replicates. Activity of compound **9** (10 and 100 μM) at hGAT1 (C), hGAT2 (D) and hGAT3 (E) transporters stably expressed in CHO cells, given as a percentage of total [^3^H]GABA uptake. Data shown are pooled experiments (*n* = 2) with three technical replicates normalized to 100% [^3^H]GABA uptake (mean ± SEM).

All selected compounds were tested for their ability to inhibit human BGT1 (hBGT1) function. An [^3^H]GABA uptake assay was performed using CHO cell lines with stable expression of the transporter, as previously described.^20^ Among all analyzed compounds, three showed clear inhibitory activity at a screening concentration of 100 μM, i.e., **3** (40% inhibition of uptake), **9** (81% inhibition), and compound **11** (26% inhibition) (Figure 3). For the most active compound **9**, [^3^H]GABA uptake assays on the other subtypes of human GABA transporters (hGAT1, hGAT2, and hGAT3) were additionally performed (Figure 3). Compound **9** at a concentration of 100 μM had no inhibitory effect at hGAT1, whereas at hGAT2 and hGAT3, it showed approximately 25 and 73% inhibition of GABA uptake, respectively. Given the significant activity of compound **9** at hBGT1 and hGAT3, the IC_50_ value was determined to be in the range of these two transporters. The IC_50_ value of compound **9** was found to be 13.9 μM for hBGT1 (4.86 ± 0.04, pIC_50_ ± SEM, *n* = 3) (Figure 3) and 58.3 μM for GAT3 (4.23 ± 0.15, pIC_50_ ± SEM, *n* = 3) (Figure S1), indicating over 4-fold preference for hBGT1 over hGAT3.

### 2.3 Binding Mode of Compound **9** at hBGT1

**Figure 4:**
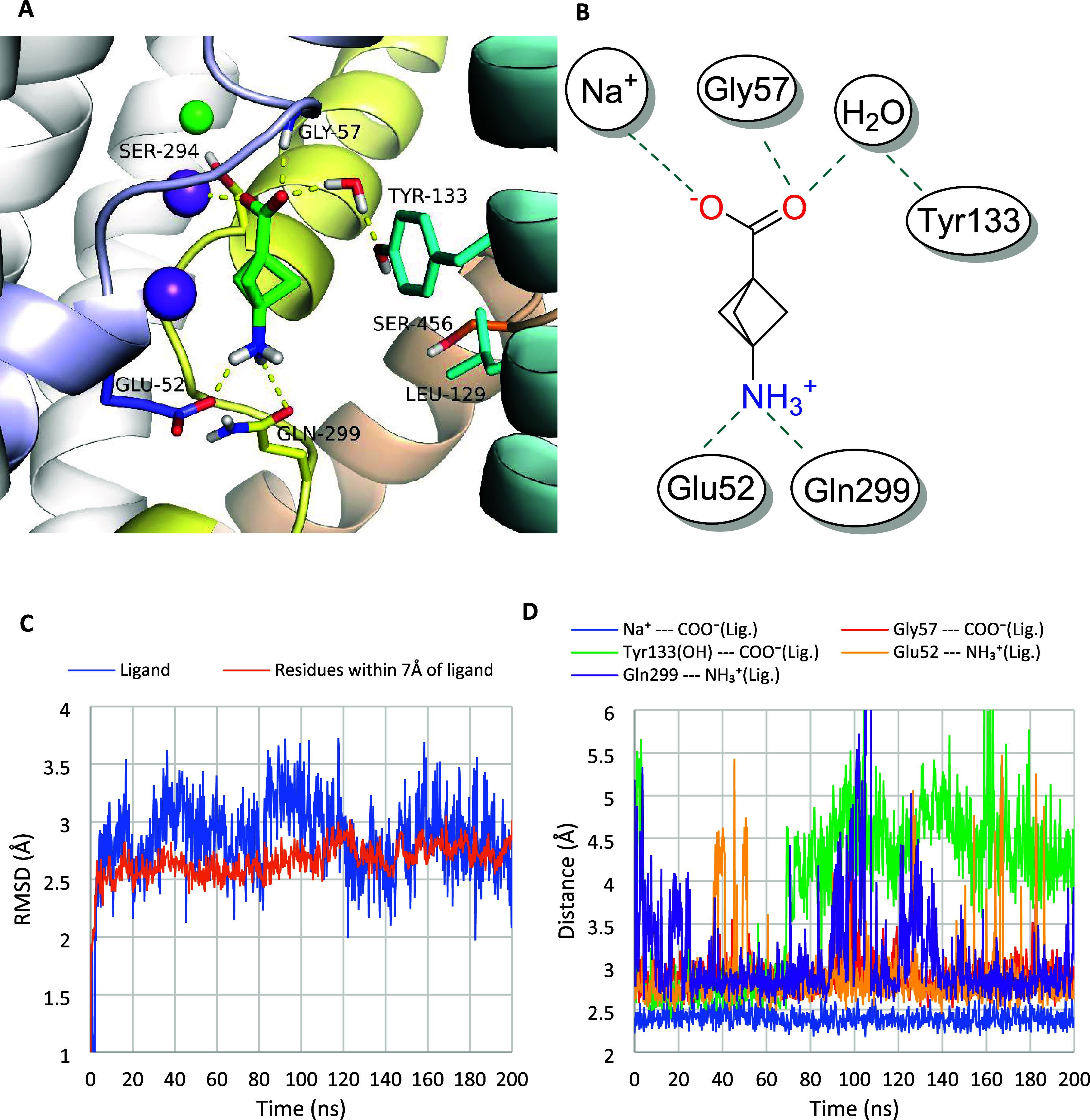
Binding mode of compound **9** within the hBGT1 transporter. The figure represents the state of ligand-transporter complex after 200 ns molecular dynamics simulation (A, B). RMSD (root-mean-square deviation) changes for compound **9** (ligand) and for residues within 7 Å of compound **9** in the course of molecular dynamics (C). Distance changes between amino acids and ligand’s functional groups involved in key interactions during MD simulation (D).

Results of docking and molecular dynamics simulations indicated that compound **9** was stably bound within the orthosteric binding site (S1) of the hBGT1 transporter (Figure 4). Its carboxyl group coordinated sodium ion Na1 and formed a hydrogen bond with Gly57. These interactions were found to be very stable during 200 ns molecular dynamics simulations (Figure 4), and they were analogous to the interactions formed by the carboxyl moiety of GABA and nipecotic acid observed in the recently revealed cryo-electron microscopy structures of hGAT1^19^ as well as leucine in the crystal structure of the leucine transporter (LeuT).^21^ In addition, MD simulations revealed the possibility of forming a water-mediated hydrogen bond with the side chain of the Tyr133. The protonated amine group of compound **9** was directed toward the bottom of the S1 site, forming a stable salt bridge with the glutamic acid residue Glu52. This amine was also located close to the amide moiety of the side chain of Gln299, which enabled the formation of a hydrogen bond as observed in the MD simulation. It is noteworthy that the Gln299 residue is specific to hBGT1, being substituted by leucine in the other GAT subtypes. Thus, the side chains of Gln299 and Glu52 created a favorable environment for the binding of the positively charged amino group of compound **9**, which could nicely explain its preferential activity on hBGT1.

### 2.4 hBGT1 Mutagenesis Studies

**Figure 5:**
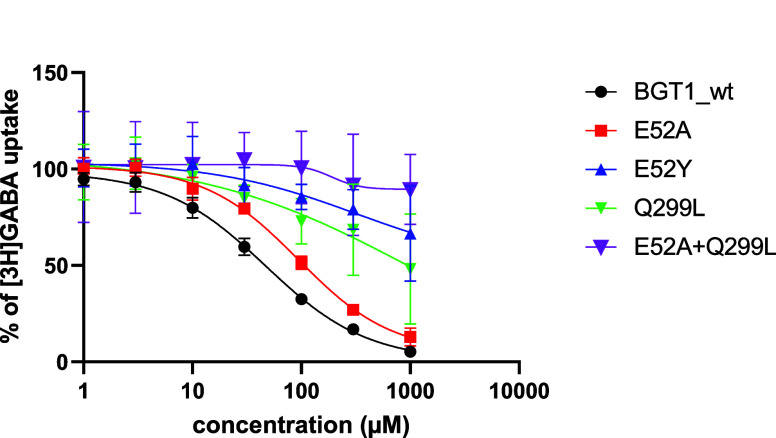
Concentration–response curves for compound **9** at the wild type (wt) of hBGT1 and mutants E52A, E52Y, Q299L, and E52A+Q299L transiently expressed in tsA201 cells. Curves were normalized to the maximal [^3^H]GABA uptake. The figure shows one representative curve for each transporter. Three to four independent experiments, performed with three technical replicates for each, gave similar results. The determined IC_50_ values are reported in Table 1.

To validate the molecular modeling, particularly the crucial interactions with the Glu52 and Gln299 residues, the activity of compound **9** was tested on the following hBGT1 mutants: E52A, E52Y, Q299L, and the double mutant E52A+Q299L (Figure 5 and Table 1).^14^ Substitution of Glu52 with alanine (E52A) resulted in an almost 2-fold decrease in the activity of the compound, while substitution with tyrosine (E52Y), as in hGAT1, led to a complete loss of activity. It appears that while the absence of the carboxyl group of glutamic acid weakened the binding of the amino group, the large aromatic tyrosine residue provided steric hindrance for the rigid compound **9**, representing the stretched conformation of GABA, and led to a complete loss of activity. This fits with the lack of compound **9** activity on GAT1. A more than 2-fold decrease in compound **9** activity was observed for the mutant in which Gln299 was replaced with leucine (Q299L), confirming the potential direct involvement of this amino acid in ligand binding. Even greater changes compared with wild type hBGT1 were observed for the E52A+Q299L double mutant, for which compound **9** showed no activity. This indicates that the predicted network of interactions between the carboxyl group of Glu52, the protonated amine of the ligand, and the amide group of Gln299 is indeed essential for the activity of compound **9**.

**Table 1:** IC_50_ Values [μM] for Compound **9** at hBGT1 and Different hBGT1 Mutants Transiently Expressed in tsA201 Cells

transporter	BGT1_wt	E52A	E52Y	Q299L	E52A + Q299L
mean IC_50_	33.9	64.2	>1000^a^	73.5	>1000^a^
pIC_50_ ± SEM	4.47 ± 0.08	4.19 ± 0.15	<3****	4.13 ± 0.05	<3****

a Curves showed less than 50% inhibition at the highest concentration (1000 μM) and were therefore not fitted. One-way ANOVA followed by Dunnett’s multiple comparison test (hBGT1 wt), significance levels *p < 0.05, **p < 0.01, ***p < 0.001, ****p < 0.0001.

It is noteworthy that the results of the mutagenesis studies for compound **9** differ to some extent from those obtained for bicyclo-GABA, which was also tested on the same hBGT1 mutants in a previous study.^12^ In the case of bicyclo-GABA, the substitution of glutamic acid Glu52 with alanine led to a drastic increase in activity, which was quite unexpected given that its predicted binding mode suggested a significant contribution of the carboxyl group of Glu52 to the binding of the protonated amine of bicyclo-GABA. The differences in activity on the E52A mutant indicate that both compounds may vary in terms of binding, kinetics, or their transport into the cell. So far, the intrinsic activity of compound **9** remains unknown.

### 2.5 GABA_A_ Receptor Binding Assay

Recognizing that compounds that are GABA analogues often display activity at GABA_A_ receptors,^12,20^ the affinity of compound **9** was assessed at native GABA_A_ receptors. To this end, a rat cortical membrane radioligand binding assay was used, according to the previously reported procedure.^22^ For the assay, [^3^H]-muscimol was used as the tritium-labeled radioligand and GABA was tested in the concentration range of 0.1 nM to 1 mM for reference. Compound **9** had no affinity for GABA_A_ receptors (at 100 μM it displaced [^3^H]-muscimol by 4%; Table 2). This distinguishes compound **9** pharmacologically from bicyclo-GABA, which is a fairly potent GABA_A_ receptor agonist (EC_50_ = 5.1 μM).^12^ Therefore, compound **9** appears to be a possible pharmacological tool to study the effects of blocking glial hBGT1/GAT3 transporters without interfering with GABA receptors.

**Table 2:** Results of Binding Assay for Compound **9** on GABA_A_ Receptors

comp.	*K*_i_ [nM] ± SEM
**9**	no affinity^a^
GABA	132.5 ± 11.5

a Compound **9** at 100 μM displaced [^3^H]-muscimol by 4%. Radioligand binding was performed using tissue rat cortex.^22^ All assays were carried out as two experiments with three replicates.

### 2.6 The Impact of Compound **9** on the Viability and Proliferation of Normal Human Astrocytes

**Figure 6:**
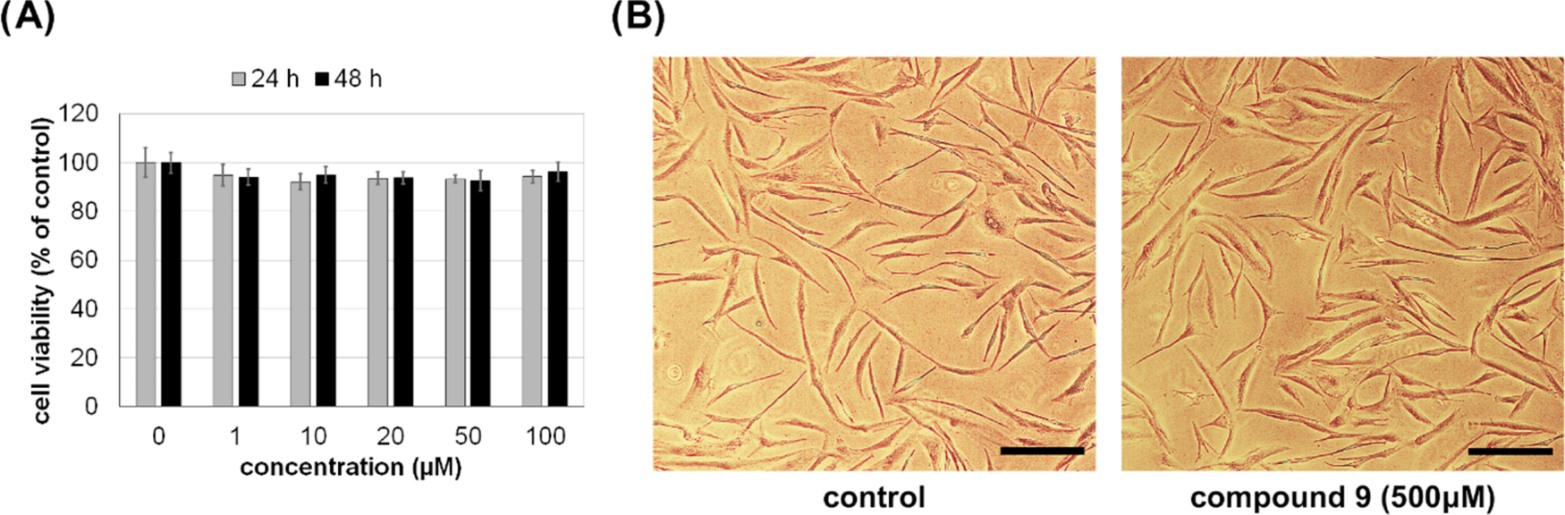
Effect of compound **9** on normal human astrocytes. (A) Viability and proliferation of NHA after the 24 or 48 h incubation with compound **9** at the concentration of 1–100 μM (solutions in culture medium) were analyzed by the WST-1 assay. Graphs represent mean ± SD of three independent experiments performed in triplicate (*n* = 3). (B) Representative images of NHA treated with 500 μM compound **9** (solution in culture medium) for 72 h. The cells were imaged by a phase-contrast microscope; scale bar = 200 μm.

Taking into account the presence of BGT1 and GAT3 transporters in astrocytes, we studied the effect of compound **9** on astrocyte viability, proliferation, and homeostasis. Normal human astrocytes (NHA) were treated with 1–100 μM compound **9** in a culture medium. The WST-1 assay was used to determine the number of metabolically active cells after 24 and 48 h of incubation. The obtained results (Figure 6a) showed that compound **9** in the concentration range 1–100 μM had no effect on astrocyte survival and proliferation. In the next step, a higher concentration (500 μM) of the compound and a longer incubation time (72 h) were applied, and the cells were examined by phase-contrast microscopy. As presented in Figure 6b, the 3-day treatment of cells with a 500 μM solution of compound **9** in a culture medium caused no changes in astrocyte morphology and confluency. Considering the results shown in Figure 6, compound **9** was not cytotoxic toward human astrocytes under *in vitro* culture conditions. These results allow compound **9** to be considered as a potential candidate for further *in vivo* studies.

### 2.7 Compound **9** and GABA *In Vitro* Effect on Normal Human Astrocyte Homeostasis

**Figure 7:**
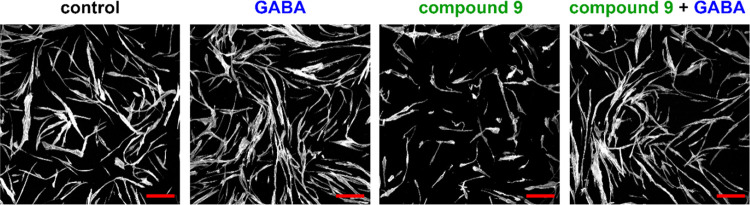
Morphological changes of astrocytes induced by compound **9** and reversal of the effect by GABA. After the 72 h incubation of NHA in PBS (control), 500 μM GABA in PBS, 500 μM compound **9** in PBS, or a solution of GABA (500 μM) and compound **9** (500 μM) in PBS, the cells were fixed and stained with Phalloidin-Atto 565. The samples were scanned using a laser confocal microscope; scale bar = 200 μm.

Using the same astrocytes, demonstrated to possess mechanisms to regulate the extracellular and intracellular levels of GABA particularly via GABA transporters,^23,24^ we next sought to investigate the effect of compound **9** on the morphology of astrocytes. To this end, we tested whether blocking the transport of GABA under experimental non-GABA extracellular conditions may impair the homeostasis of these cells. Due to the fact that the normal human astrocyte culture medium provides cells with GABA, an experimental model based on the incubation of cells in phosphate-buffered saline (PBS) with Ca^2+^ and Mg^2+^ was used. Therefore, the cells were incubated for 72 h in PBS (control), 500 μM GABA in PBS, 500 μM compound **9** in PBS, or a solution of GABA (500 μM) and compound **9** (500 μM) in PBS. Afterward, the cells were stained to visualize actin filaments using confocal microscopy. As shown in Figure 7, the treatment of cells with compound **9** in a non-GABA environment resulted in cell shrinkage and a loss of cell-to-cell contact. GABA prevented these changes, suggesting that the effect of compound **9** on normal human astrocytes may be due to the inhibition of GABA turnover. It should be noted that the same compound **9** concentration (500 μM) and incubation time (72 h), but in the presence of a culture medium, did not cause changes in astrocyte morphology (Figure 6b). Furthermore, this may also suggest a competitive inhibition mode for compound **9** where a high concentration of GABA reverses the effect of the inhibitor. Nevertheless, further kinetic studies are necessary to verify this hypothesis.

### 2.8 Neuroprotective Activity of Compound **9**

**Figure 8:**
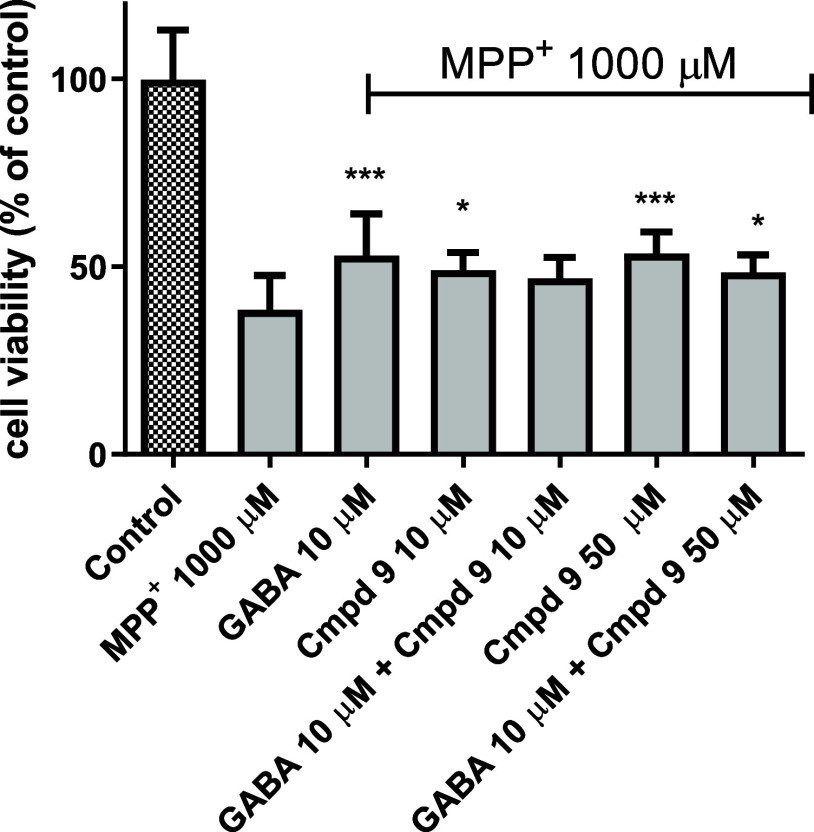
Effect of compound **9** at 10 or 50 μM and/or GABA at 10 μM on SH-SY5Y neuroblastoma cell viability damaged by 1 mM MPP^+^ after 24 h of incubation. Graphs represent mean ± SD of two independent experiments performed in quadruplicate. Statistical significance was set at ***p < 0.001, **p < 0.01, *p < 0.05 by GraphPad Prism 8 software using one-way ANOVA and Bonferroni’s post hoc test in comparison with the positive control MPP^+^ (1000 μM).

Numerous studies have reported the important role of GABA as neuroprotective agent against degeneration induced by toxins or injury following cerebral ischemia.^3,22^ As compound **9** is a BGT1/GAT3 inhibitor with a preference for BGT1 and thus would facilitate increases in the GABA levels, we aimed to examine its neuroprotective activity using undifferentiated SH-SY5Y neuroblastoma cells. The model used mimics certain aspects of neurodegenerative diseases, where GABAergic dysfunction and neuronal death are prominent features. Because compound **9** showed no impact on SH-SY5Y cell line viability (Figure S2), its potential neuroprotective activity was therefore assessed using GABA as a positive control. The neuroprotection studies were performed to determine the ability of compound **9** to prevent MPP^+^ (1-methyl-4-phenylpyridinium) toxicity at a concentration of 1 mM, which was coincubated with compound **9** for 24 h. MPP^+^ is a well-established neurotoxin that selectively damages neurons, making it a commonly used agent to model aspects of neurodegenerative conditions *in vitro*. It induces oxidative stress and mitochondrial dysfunction, leading to cell death. Cell viability was detected by a standard MTS assay. The neurotoxin MPP^+^ significantly reduced the viability of the SH-SY5Y cells. Separate application of GABA (10 μM) or compound **9** (10 or 50 μM) increased cell viability compared to the control with MPP^+^ alone. However, we found that while both GABA (10 μM) and compound **9** (50 μM) alone significantly increased cell viability compared to MPP^+^ treatment, the coapplication of GABA and compound **9** (50 μM) showed a slightly reduced effect compared to either compound applied separately (Figure 8).

## 3 Conclusions

Application of multilevel virtual screening led to the identification of compound **9**, an inhibitor with a preference for the BGT1 transporter. This compound revealed also inhibitory activity against GAT3 while it was inactive or only weakly active toward other GABA transporters, thus making it a glial-selective GAT inhibitor. *In silico* experiments predicted the binding mode of compound **9**, which was confirmed by mutagenesis studies. It was shown that interactions with Glu52 and Gln299 are important for BGT1 transporter inhibition, as compound **9** was less active or completely inactive at E52A, E52Y, Q299L, and E52A + Q299L mutants. Subsequent evaluation showed that compound **9** had no affinity for GABA_A_ receptors. Moreover, it was found to be nontoxic when tested on normal human astrocytes and showed some potential for neuroprotective activity on SH-SY5Y cells. The identified hit compound appears to be a promising scaffold for the development of novel derivatives with the selective inhibition of the BGT1 transporter. These compounds could serve as valuable tools for investigating the role of BGT1 in the CNS, particularly in relation to its involvement in GABA regulation and neuroprotection. While the role of BGT1 in epilepsy remains uncertain, further studies using these compounds could provide important insights into BGT1 contribution to CNS function and help clarify its potential as a therapeutic target.

## 4 Methods

### 4.1 Virtual Screening

From the ZINC15 database, tranches of 3D compound structures displaying desired physicochemical characteristics (molecular weight (MW) < 200 and *c* log *P* < 1) were selected and downloaded in SDF format. In the case of Enamine screening collection in SDF format, compounds were filtered based on preferred molecular weight and cLogP values using the Ligand Filtering functionality of Maestro 12.5 software. Pharmacophore model was built with LigandScout 4.4.6 program and was based on known hBGT1 ligands with amino acid structure. This model employed a merged feature pharmacophore approach, incorporating pharmacophore fit and atom overlap scoring function. The most efficient conformer generation was applied to both pharmacophore generation and screening process. During the screening, the best matching conformation for each compound was retrieved. Match all query features mode was used. The screening allowed for up to three features to be omitted, while exclusion volumes were not taken into account. The homology model of hBGT1 was built using the SWISS-MODEL web server based on the crystal structure of dopamine transporter (PDB code: 4XPA) and default alignment generated by SWISS-MODEL. The model was devoid of N- and C-termini due to the low sequence similarity between hBGT1 and DAT. The sodium and chloride ions from the template were retained. Before molecular docking, the model was prepared with Protein Preparation Wizard available in Maestro software, using default settings. All compounds were optimized with LigPrep in the OPLS3e force field, and the ionization states were predicted with Epik v5.3 for pH = 7.4 ± 0.5. Compounds from ZINC15 and Enamine were docked to the hBGT1 model using the Glide v8.8 program. The grid center was defined as a centroid of residues Tyr133, Phe293, Gln299, Glu52, and Gly57. The inner box size was set to 15A x 15A x 15A. Docking was performed in the OPLS3e force field with Extra Precision.

### 4.2 Molecular Dynamics Simulations

The input files for the molecular dynamics were prepared with the System Builder module of the Schrödinger Suite. A TIP3P water type and a POPC(300 K) membrane model were applied. The model structure prealigned with the OPM server was placed in a membrane. The size of the orthorhombic box was calculated according to the buffer method. The buffer distance between the protein model and the simulation box boundary was set as follows: *a* = 12 Å, *b* = 12 Å, and *c* = 10 Å. The system was neutralized by adding an appropriate number of chloride ions. Then, 0.15 M NaCl was added to provide the standard physiological ionic strength. MD simulations in DESMOND v6.3 were run in an NPT ensemble at 300 K with a time step of 2 fs. The total duration of the simulation was 200 ns with a recording interval of 50 ps. The system was relaxed before the simulation according to the default six-step DESMOND protocol. A seed was set as random, and the other options were default. During the system setups, as well as the simulations, the OPLS3e force field was applied. RMSD and interactions were analyzed in VMD 1.9.3 program. Binding mode was visualized in the PyMOL 2.4.1 program.

### 4.3 Tested Compounds

Compounds selected from the virtual screening were purchased from two suppliers: Molport (**1**–**8**, **10**, and **12**) and Chemspace (**9**, **11**, and **13**). Their supplier IDs were as follows: MolPort-044-747-180, MolPort-045-971-495, MolPort-019-930-938, MolPort-038-073-734, MolPort-023-245-437, MolPort-042-620-851, MolPort-047-920-563, MolPort-028-600-683, MolPort-019-931-102, MolPort-020-097-263, CSSB00000761587, CSSS00132302455, and CSSS06256032673, respectively. The most active compound **9** can be purchased directly from Enamine (ID: EN300-88073). All compounds, except **4**, were tested as the hydrochlorides. The purity of the samples was >90% (**1**–**6**, **8**, **11**–**13**) or >95% (**7**, **9**, **10**).

### 4.4 [^3^H]GABA Uptake Assay

The [^3^H]GABA uptake assay was performed as previously described using the CHO Flp-In cell line (including culturing conditions) exhibiting stable expression of GABA transporters.^20,25^ The CHO cells were placed in 96-well plates (approximately 50,000 cells/well) for about 24 h before the experiment. For the experiment, cells were incubated for 3 min in the presence of a 30 nM solution of [2,3-^3^H(N)]GABA ([^3^H]GABA; specific radioactivity 35.0 Ci/mmol) and two concentrations of test compounds (10 and 100 μM). The maximum uptake was determined based on incubation of cells with the 30 nM solution of [^3^H]GABA alone, and nonspecific binding was determined through incubation with a 30 nM solution of [^3^H]GABA in the presence of a 3 mM solution of unlabeled GABA. After incubation, cells were washed 3 times with 100 μL/well ice-cold assay buffer, 150 μL/well MicroScint20 (PerkinElmer) was subsequently added before the plate was shaken for at least 1 h. The radioactivity of each well of the plate was measured on a TopCount NXT scintillation counter. The [^3^H]GABA uptake data were normalized to the total uptake. Data presented are the pooled data of at least three independent experiments with three technical replicates if not stated otherwise in figure legends. Concentration–response curves were fitted by nonlinear regression to the sigmoidal concentration–response model with GraphPad Prism v 9.0.

### 4.5 hBGT1 Mutational Studies

The tsA201 cell line (a transformed HEK293 cell line) has been described previously and was cultured accordingly.^20^ The tsA201 cells used for transient expression of hBGT1 mutants were transfected with DNA constructs (8 μg per 10 cm plate) using 40 μL of PolyFect transfection reagent according to the manufacturer’s protocol (Qiagen). The mutated DNA constructs E52Y, E52A, and Q299L + E52A were synthesized by Genscript (Piscataway, NJ),^14^ while the Q299L mutation was introduced in-house into HA-tagged hBGT1 using the QuikChange II site-directed mutagenesis kit (Stratagene, La Jolla, CA)^26^ and subsequently verified by sequencing. The [^3^H]GABA uptake assay for hBGT1 mutants was carried out according to the same procedure as that for wild type GATs.

### 4.6 GABA_A_ Receptor Binding Assay

A 10 mM stock solution of the tested compound was prepared in DMSO. Serial dilutions of the compound were prepared in 96-well microplates in assay buffers using automated pipetting system epMotion 5070 (Eppendorf). Each compound was tested in 8 concentrations from 10^–3^ to 10^–10^ M (final concentration). Radioligand binding was performed using tissue rat cortex according to the previous procedure.^22^ All assays were carried out in duplicates. 50 μL of working solution of the tested compounds, 50 μL of [^3^H]-muscimol (final concentration 2.0 nM), and 150 μL of tissue suspension prepared in assay buffer (50 mM Tris-HCl, pH 7.4) were transferred to polypropylene 96-well microplate using 96-well pipetting station Rainin Liquidator (Mettler-Toledo). GABA (100 μM) was used to define nonspecific binding. The microplate was covered with sealing tape, mixed, and incubated for 15 min at 4 °C. The reaction was terminated by rapid filtration through GF/B filter mate. Ten rapid washes with 200 μL of 50 mM Tris buffer (4 °C, pH 7.4) were performed using 96-well FilterMate harvester (PerkinElmer). The filter mates were dried at 37 °C in forced air fan incubator and then solid scintillator MeltiLex was melted on filter mates at 90 °C for 4 min. The radioactivity on the filter was measured in MicroBeta TriLux 1450 scintillation counter (PerkinElmer). Data were fitted to a one-site curve-fitting equation with Prism 8.0 (GraphPad Software), and *K*_i_ values were estimated from the Cheng–Prusoff equation.

### 4.7 *In Vitro* Studies on Normal Human Astrocytes

#### 4.7.1 Cell Culture

Gibco Human Astrocytes (Thermo Fisher Scientific) were maintained in a humidified 5% CO_2_ incubator at 37 °C. The cells were cultured in Gibco Astrocyte Medium (DMEM with GlutaMAX, N-2 Supplement, and OneShot Fetal Bovine Serum) supplemented with penicillin G (100 U/mL), neomycin (10 μg/mL), and amphotericin B (0.25 μg/mL).

#### 4.7.2 WST-1 Assay

The effect of compound **9** on cell viability and cell proliferation was evaluated using a WST-1 assay (Roche GmbH, Mannheim, Germany) based on the cleavage of the WST-1 reagent into formazan by mitochondrial dehydrogenases in metabolically active (viable) cells. In brief, NHA in the 96-well microplate were treated with compound **9** solutions (1–100 μM) or incubated in the culture medium (control). After 24 or 48 h, the WST-1 (10 μL) was added to each well for 3 h. The absorbance was measured at 440 and 650 nm using a microplate reader Infinite 200 PRO (TECAN).

#### 4.7.3 Confocal Imaging

NHA was grown on a coverslip in a 35 mm culture dish. After treatment, the cells were fixed (4% paraformaldehyde) and permeabilized (0.1% Triton X-100). Then, the samples were stained with Phalloidin-Atto 565 (Sigma-Aldrich, Inc.) according to the manufacturer’s protocol. Laser confocal microscope Nikon Eclipse Ti-E A1R-Si and Nikon NIS Elements AR software were used for cell imaging.

#### 4.7.4 Statistical Analysis

Statistical analysis was performed using GraphPad Prism 8.0.1 software. Differences between groups were assessed by one-way ANOVA and Dunnett’s multiple comparison test. A *p*-value <0.05 was used as the cutoff for statistical significance.

### 4.8 Neuroprotective Activity Testing

Neuroprotection studies were performed according to the procedure described previously.^27^ In brief, SH-SY5Y CRL-2266 (American Type Culture Collection, Manassas, VG) were seeded in microplate at a concentration of 2.5 × 10^4^ cells/well in 100 μL of culture medium and cultured for 24 h at 37 °C and 5% CO_2_. The cells were preincubated first for 1 h with compound **9** (10 or 50 μM) and/or GABA (10 μM). Next, MPP^+^ was added at a final concentration of 1 mM and the cells were placed in the incubator. After 24 h of the compound coincubation with MPP^+^, the CellTiter 96 AQueous Nonradioactive Cell Proliferation Assay (MTS) was performed. The test was purchased from Promega (Madison, WI). The absorbance was measured using a microplate reader EnSpire (PerkinElmer, Waltham, MA) at 490 nm. All results were shown as mean ± SD. The statistical significances were calculated by GraphPad Prism 6.0 software.

## Supporting Information Available

List of compounds used to construct the pharmacophore model (S1), IC_50_ determination at GAT3 (S2), and the impact of compound **9** on the viability of SH-SY5Y neuroblastoma cells (S3) (PDF)
