# Supplementary material for: Rational Search for Betaine/GABA Transporter 1 Inhibitors—In Vitro Evaluation of Selected Hit Compound
Source: ACS Chem Neurosci. 2024 Oct 19;15(21):4046–54. doi: 10.1021/acschemneuro.4c00425 (PMC11587516; doi:10.1021/acschemneuro.4c00425)
Supplement: Supplementary file 1 — cn4c00425_si_001.pdf [file cn4c00425_si_001.pdf]

Supplementary information

Rational search for betaine/GABA transporter 1 inhibitors – *in vitro* evaluation of selected hit compound

Kamil Łątka[1], Stefanie Kickinger[2], Zuzanna Rzepka[3], Paula Zaręba[1], Gniewomir Latacz[4], Agata Siwek[5], Małgorzata Wolak[5], Dorota Stary[1], Monika Marcinkowska[6], Petrine Wellendorph[2], Dorota Wrześniok[3], Marek Bajda[1,*]

[1] Department of Physicochemical Drug Analysis, Jagiellonian University Medical College, Medyczna 9, 30-688 Krakow, Poland
[2] Department of Drug Design and Pharmacology, Faculty of Health and Medical Sciences, University of Copenhagen, 2100 Copenhagen, Denmark
[3] Department of Pharmaceutical Chemistry, Faculty of Pharmaceutical Sciences in Sosnowiec, Medical University of Silesia, Jagiellońska 4, 41-200 Sosnowiec, Poland
[4] Department of Technology and Biotechnology of Drugs, Jagiellonian University Medical College, Medyczna 9, 30-688 Kraków, Poland
[5] Department of Pharmacobiology, Faculty of Pharmacy, Jagiellonian University Medical College, Medyczna 9, 30-688 Kraków, Poland
[6] Department of Medicinal Chemistry, Jagiellonian University Medical College, Medyczna 9, 30-688 Krakow, Poland
Corresponding author: marek.bajda@uj.edu.pl

S1. List of compounds used to construct the pharmacophore model

S1.1. Active compounds (BGT1 inhibitors)

N[C@]12[C@@H](C2)[C@H](CC1)C(=O)O
O=C(O)C[C@H]1[C@H](N)C1
NC1=NC[C@H](C(=O)O)CC1
NC1=NC[C@@H](C(=O)O)CC1
NC1=NC[C@@H](C(=O)O)CN1

S1.2. Compounds with low activity or inactive

NC1=NC=CC(=C1)C(O)=O
OC1=NOC2=C1CCNC2
OC1=NOC2=C1CNCC2
N[C@H]1CCCC2=C1C(O)=NO2
OC1=NOC2=C1CNCCC2
NCC1=CC(O)=NO1
NC(=N)NCC1=CC(O)=NO1
NC1=NC(CC(O)=O)=CS1
OC(=O)C1=CC=CC2=C1N=CN2
OC(=O)C1=CC2=C(NC=N2)C=C1
OC(=O)[C@@H]1CCC2=C(C1)N=CN2
OC(=O)[C@@H]1CCCC2=C1N=CN2
OC(=O)C[C@H]1CCCN1
CC(C)([C@H]1CCCN1)C(O)=O
C[C@H]([C@H]1CCCN1)C(O)=O
O[C@@H]([C@H]1CCCN1)C(O)=O
N[C@H]1CC=C(C1)C(O)=O
N[C@H]1CC[C@@H](C1)C(O)=O
N[C@@H]1C[C@H](C=C1)C(O)=O
OC(=O)[C@H]1CCNC1
N[C@H]1CCC[C@H](C1)C(O)=O
N[C@H]1CCCC[C@@H]1C(O)=O
OS(=O)(=O)[C@@H]1CCCNC1
OC(=O)[C@@H]1CCCCNC1
OC(=O)C1=CCCCNC1
OC(=O)[C@H]1CCCNC1
OC(=O)C1=CCCNC1
C[C@H]1CNCC(=C1)C(O)=O
O[C@@H]1CNC[C@@H](C1)C(O)=O
N[C@H]1CCNC[C@@H]1C(O)=O
O[C@H]1CCNC[C@@H]1C(O)=O
C[C@@H]1CNC[C@@H]([C@H]1O)C(O)=O

OC(=O)[C@H]1CNCC[C@@H]1S
OC(=O)C1CCNCC1
OC(=O)C1=CCNCC1
O[C@@H]1CNCC[C@H]1C(O)=O
OC(=O)CCCC1=NC=CN1
OC(=O)\C=C\C1=NC=CN1
OC(=O)\C=C\C1=CNC=N1
OC(=O)\C=C\C1=CN=CN1
OC(=O)CCC1=CNC=N1
OC(=O)CCC1=CN=CN1
OC(=O)CCN1C=CN=C1
OC(=O)CCCC1=NC=CN1
OC(=O)CCCCCCN1C=CN=C1
N[C@]12C[C@H]1[C@@H](CC2)C(O)=O
N[C@H]1C[C@@H]1CC(O)=O
N[C@@H]1C[C@@H]1CC(O)=O
N[C@H]1C[C@H]1CC(O)=O
NC[C@H]1C[C@H]1C(O)=O
NC[C@@H]1C[C@@H]1C(O)=O
NC[C@@H]1C[C@H]1C(O)=O
NC[C@H]1C[C@@H]1C(O)=O
NC1=CC=C(C=N1)C(O)=O
NC1=NC=C(C=N1)C(O)=O
NC(=N)N1CCC(CC1)C(O)=O

S2. IC_{50} determination at GAT3

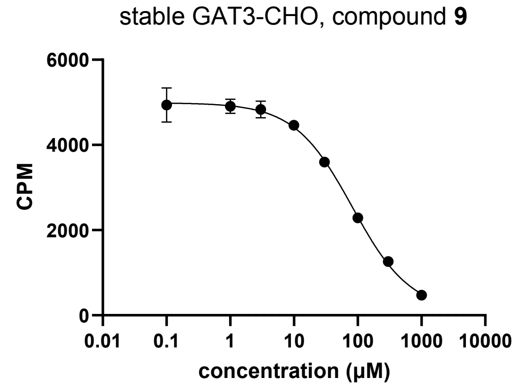

Figure S1. IC_{50} determination for compound **9**. Data represent one of three independent experiments, each performed in three technical replicates.

S3. The impact of compound 9 on the viability of SH-SY5Y cells

Before testing neuroprotective activity of compound **9** on SH-SY5Y neuroblastoma cell lines the impact of the compound on cell viability was tested as described below.

S3.1. Results and discussion

The influence of compound **9** on viability of SH-SY5Y neuroblastoma cells was determined using Aqueous Non-Radioactive Cell Proliferation Assay. The cells were treated with different solutions (0.1-100 µM) of compound **9** or doxorubicin (DOX, 1 µM), which is the reference cytostatic drug, and incubated for 72h. Upon adding MTS and following 2h incubation, cell viability was calculated and compared. It was shown that doxorubicin significantly decreased SH-SY5Y viability, while the samples with the tested compound were at the level of control (**Figure S2**). As compound **9** showed no impact on neuroblastoma viability, it could be tested further regarding its potential neuroprotective activity.

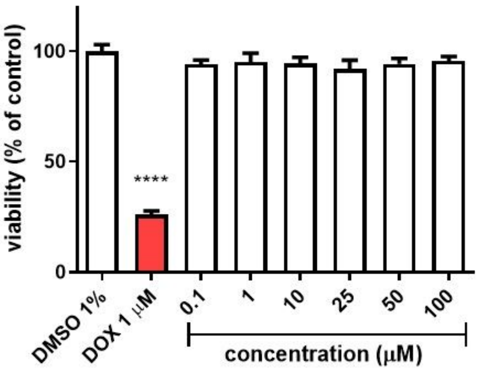

Figure S2. The effect of compound **9** at different concentrations (0.1-100 μM) on SH-SY5Y neuroblastoma cell viability. Cytotoxic doxorubicin was used as the reference compound at concentration of 1 μM. Graphs represent means ± SD of two independent experiments performed in quadruplicate. Statistical significance was set at $****p < 0.0001$, $***p < 0.001$, $**p < 0.01$, $*p < 0.05$ by GraphPad PrismTM 8 software using One-way ANOVA and Bonferroni's post-hoc test.

S3.2. Methods

The effect of compound **9** on viability and proliferation of SH-SY5Y was determined with CellTiter 96® Aqueous Non-Radioactive Cell Proliferation Assay (MTS, Promega, Madison, WI, USA). The cells were treated with compound **9** solutions (0.1-100 μM) or the reference cytostatic drug doxorubicin (DOX, 1 μM) and incubated for 72h. Next MTS was added in fresh medium and after 2h of incubation the absorbance was measured at 490 nm using a microplate reader EnSpire (PerkinElmer, Waltham, MA USA). All results were shown as mean ± SD calculated from two independent experiments. The statistical significances were calculated by GraphPad Prism 6.0 software.
